# Supplementary material for: Dynamic Localization of Paraspeckle Components under Osmotic Stress
Source: Noncoding RNA. 2024 Apr 12;10(2):23. doi: 10.3390/ncrna10020023 (PMC11053584; doi:10.3390/ncrna10020023)
Supplement: Supplementary file 1 [file ncrna-10-00023-s001.zip › ncrna-2935253-supplementary.pdf]

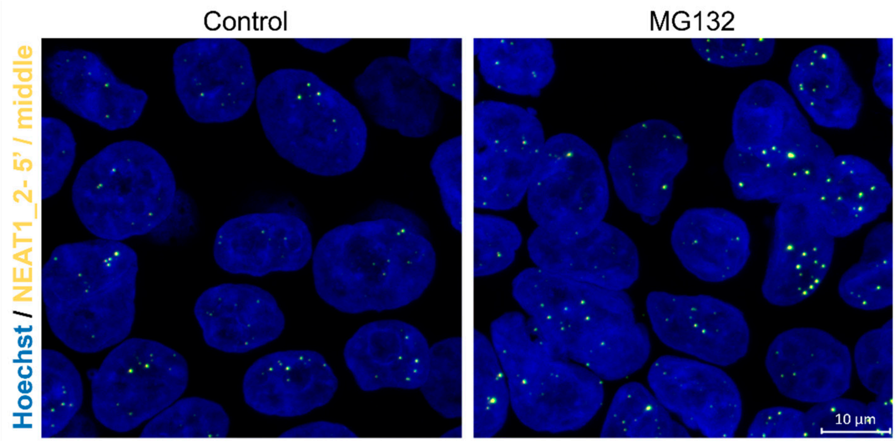

Supplementary Figure S1. Proteasome Inhibitor MG132 increases NEAT1_2 in HEK293T cells. Proteasome inhibition was used as a reference for stress experiments because it is known to increase NEAT1_2 expression. After 4hrs of MG132 treatment, an apparent increase was observed in nuclear NEAT1_2 foci with smFISH experiments.

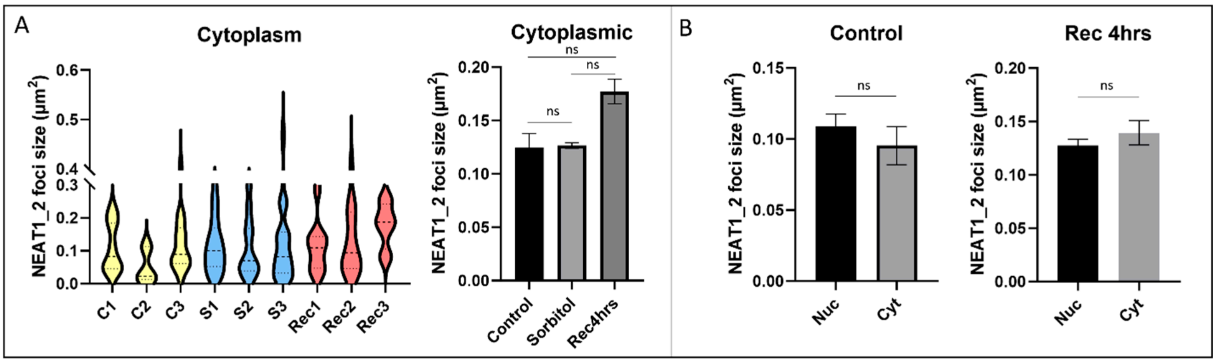

Supplementary Figure S2. Size of NEAT1_2 foci in cytoplasm shows no difference between conditions of control, osmotic stress and recovery. (A) No significant difference was observed in size of cytoplasmic NEAT1_2 foci between different conditions. (B) Size of NEAT1_2 foci in the nucleus and the cytoplasm show no difference under conditions of control and recovery.

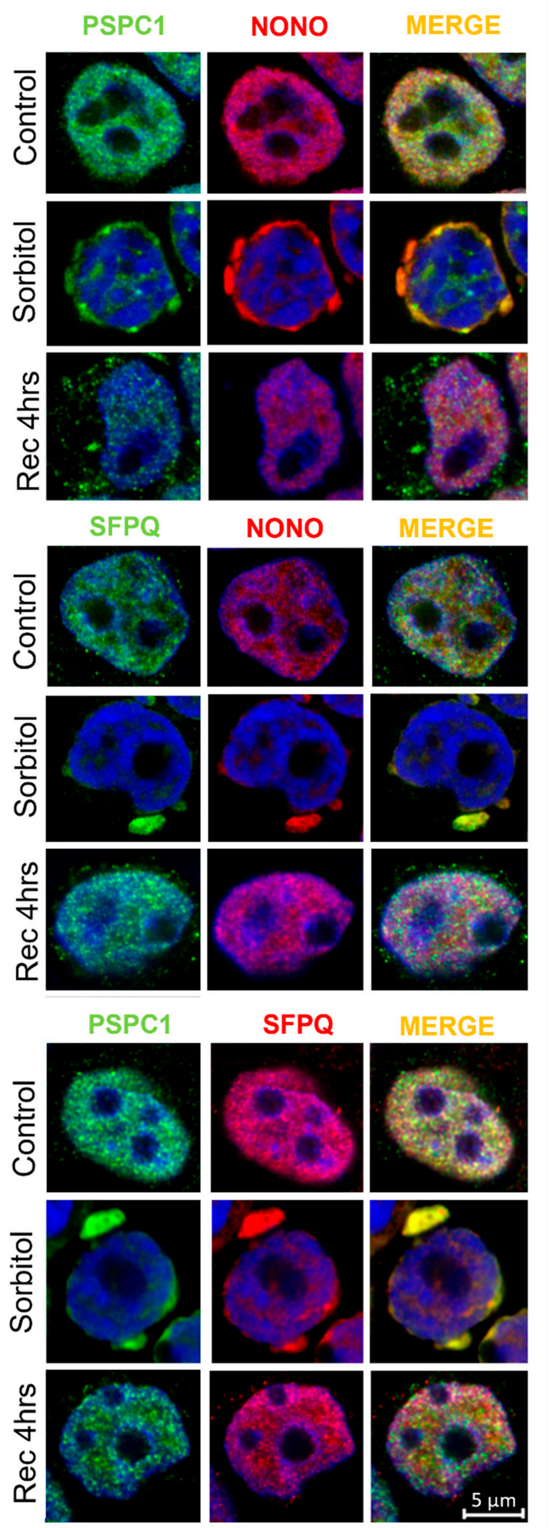

Supplementary Figure S3. Extended data for Figure 4. Separated channels show the individual localization of PSPC1, NONO, and SFPQ in HEK293T cells under conditions of control, osmotic stress, and recovery. Osmotic stress induces the formation of cytoplasmic aggregation of PSPs together.

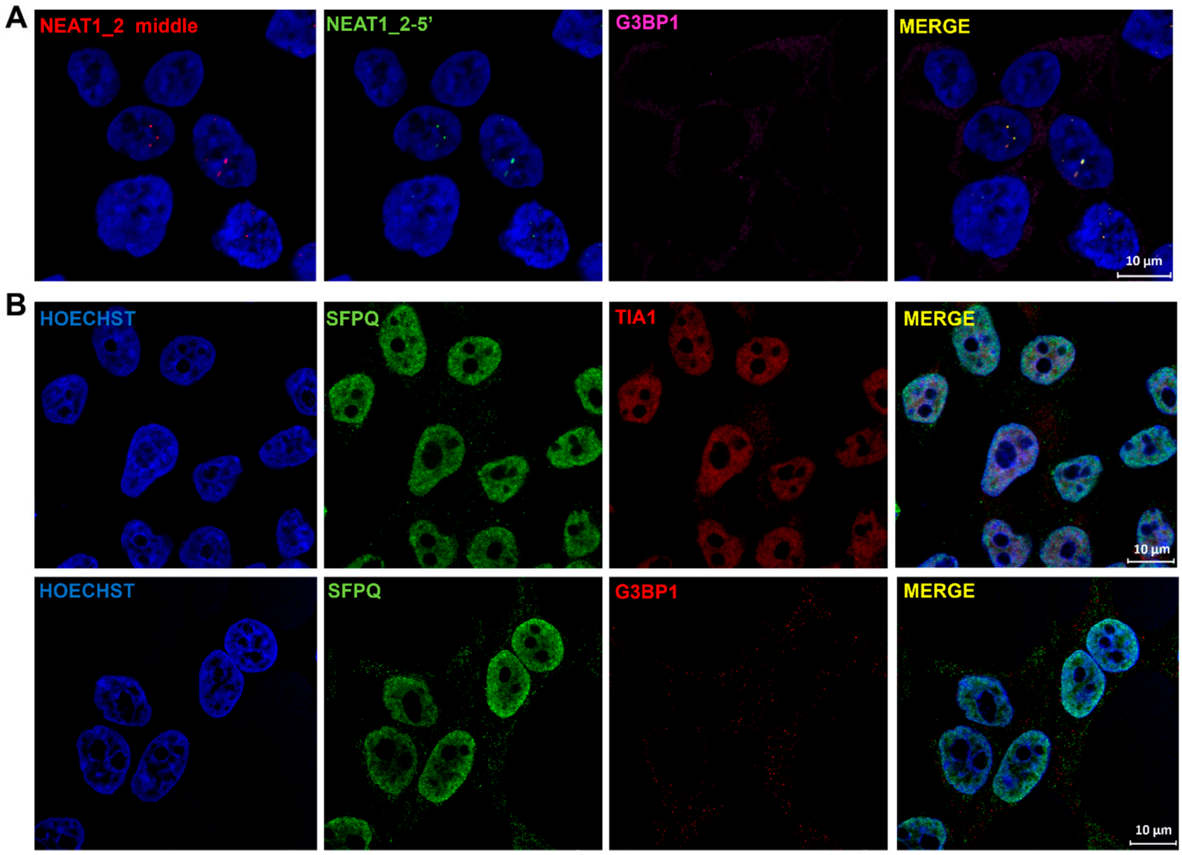

Supplementary Figure S4. Subcellular localization of SG markers under control conditions. No SG formation and colocalization of SG markers with NEAT1_2 (A) and PSPs (B) were observed in the cytoplasm in the control samples.

Supplementary Table S1. Size distribution of NEAT1_2 foci in groups and subcellular compartments

	Mean		Range		IQR*	
	Nuc	Cyt	Nuc	Cyt	Nuc	Cyt
**Control**	0.108	0.125	1.071	0.378	0.108	0.118
**Sorbitol**	0.061	0.127	0.462	0.465	0.078	0.120
**Rec4hrs**	0.128	0.177	0.964	0.377	0.129	0.141

*IQR: Interquartile range
